# In Vitro Evaluation of Polihexanide, Octenidine and NaClO/HClO-Based Antiseptics against Biofilm Formed by Wound Pathogens

**DOI:** 10.3390/membranes11010062

**Published:** 2021-01-17

**Authors:** Grzegorz Krasowski, Adam Junka, Justyna Paleczny, Joanna Czajkowska, Elżbieta Makomaska-Szaroszyk, Grzegorz Chodaczek, Michał Majkowski, Paweł Migdał, Karol Fijałkowski, Beata Kowalska-Krochmal, Marzenna Bartoszewicz

**Affiliations:** 1 Nutrikon, KCZ Surgical Ward, 47-300 Krapkowice, Poland; g.krasowski@wp.pl; 2 Department of Pharmaceutical Microbiology and Parasitology, Faculty of Pharmacy, Wrocław Medical University, 50-556 Wrocław, Poland; justyna.paleczny@student.umed.wroc.pl (J.P.); beata.kowalska-krochmal@umed.wroc.pl (B.K.-K.); marzenna.bartoszewicz@umed.wroc.pl (M.B.); 3 Laboratory of Microbiology, Łukasiewicz Research Network—PORT Polish Center for Technology Development, 54-066 Wrocław, Poland; joanna.czajkowska@port.org.pl; 4 Faculty of Medicine, Lazarski University, 02-662 Warszawa, Poland; elzbieta.makomaska-szaroszyk@lazarski.pl; 5 Bioimaging Laboratory, Łukasiewicz Research Network—PORT Polish Center for Technology Development, 54-066 Wrocław, Poland; grzegorz.chodaczek@port.org.pl (G.C.); michal.majkowski@port.org.pl (M.M.); 6 Department of Environment Hygiene and Animal Welfare, Wroclaw University of Environmental and Life Sciences, 51-630 Wrocław, Poland; pawel.migdal@upwr.pl; 7 Department of Microbiology and Biotechnology, Faculty of Biotechnology and Animal Husbandry, West Pomeranian University of Technology, 70-311 Szczecin, Poland; karol.fijalkowski@zut.edu.pl

**Keywords:** wound biofilm, polihexanide, octenidine, hypochlorous acid, sodium hypochlorite

## Abstract

Chronic wounds complicated with biofilm formed by pathogens remain one of the most significant challenges of contemporary medicine. The application of topical antiseptic solutions against wound biofilm has been gaining increasing interest among clinical practitioners and scientific researchers. This paper compares the activity of polyhexanide-, octenidine- and hypochlorite/hypochlorous acid-based antiseptics against biofilm formed by clinical strains of *Candida albicans, Staphylococcus aureus* and *Pseudomonas aeruginosa*. The analyses included both standard techniques utilizing polystyrene plates and self-designed biocellulose-based models in which a biofilm formed by pathogens was formed on an elastic, fibrinous surface covered with a fibroblast layer. The obtained results show high antibiofilm activity of polihexanide- and octenidine-based antiseptics and lack or weak antibiofilm activity of hypochlorite-based antiseptic of total chlorine content equal to 80 parts per million. The data presented in this paper indicate that polihexanide- or octenidine-based antiseptics are highly useful in the treatment of biofilm, while hypochlorite-based antiseptics with low chlorine content may be applied for wound rinsing but not when specific antibiofilm activity is required.

## 1. Introduction

Biofilm is a community of microorganisms embedded within an extracellular, protective coating [1]. Biofilm may be attached to the abiotic/biotic surface or may float at the liquid–air interface. Although biofilm is a predominant form of microbial existence, its significance, especially in the context of human health and disease, was recognized barely a few decades ago [2]. Among the main reasons were the disadvantages of traditional microbial sampling techniques which disrupt the spatial organization of biofilm-forming microorganisms [3]. Since that time, the development of analytical techniques, including advanced microscopic methods, has allowed the identification of a vast number of pathological, disease entities referred to as biofilm-related infections (BRI). The most prominent examples of such infections include those occurring within the lungs, oral cavity, bones, medical devices, prostheses and chronic wounds [4,5,6]. A fully-grown biofilm consists of multiple microbial cells (of the same or different species) and external polymeric matrix, which significantly hinders the ability of the immune system and of drugs to penetrate throughout the structure. Moreover, due to a plethora of factors [7], biofilms contain regions with low metabolic activity [8]. This fact strongly reduces the antimicrobial potential of these of drugs which act by repressing cell division and replication. Reports indicate that biofilm-forming microorganisms may be a few hundred times more resistant to antibiotics than their free-floating (referred to also as planktonic) counterparts [9]. Because of their size, persistence and the patients’ comorbidities, chronic wounds are at particular risk of developing BRIs. The report by Bjarnsholt et al. [10] showed the presence of biofilm in 78.2% of chronic wounds with a disturbed healing pattern. If not adequately treated, such biofilm-caused, local chronic wound infections may lead to limb amputation or may develop into a systemic, life-threating disease. Therefore, infected wounds are still one of the greatest and still unsolved challenges of medicine. Because locally-delivered antibiotics are considered ineffective or inadvisable in fighting wound biofilm, other countermeasures are presently used in clinical routine. They are mainly: surgical debridement, maggot therapy, antimicrobial dressings and the application of antiseptics [11].

The latter, depending on wound/infection specifics, are frequently used together with debridement and antimicrobial dressings. Contrary to antibiotics, antiseptics’ mechanism of action is referred to as “non-specific” and their antimicrobial activity relies on the ability to destroy and to denature microbial proteins or to disrupt microbial cell walls resulting in cell death. Polyhexamethylene biguanide (PHMB) is considered one of the most effective antiseptics as regards antimicrobial spectrum of activity and biocompatibility with the patient’s tissue. PHMB molecules are of basic character and interact with acidic phospholipids of microbial membranes leading to loss of integrity and death of the microorganism. The antimicrobial spectrum of PHMB includes Gram-positive and Gram-negative bacteria, spore-forming bacteria (but not bacterial spores), such intracellular bacteria as chlamydiae and mycoplasma, and fungi including *Candida* spp. and *Aspergillus* spp [12]. Moreover, reports have shown that PHMB is well tolerated when applied on intact skin, wounds or even eyes [13]. Another potent antiseptic used, among others, to decolonize or treat infected wounds, is octenidine dihydrochloride (OCT). Being positively charged, it adheres to the negatively-charged cell walls of microorganisms, attacking enzymatic systems there and, similarly to PHMB, leading to leakage of the cytoplasmic membrane and microbial cell death. OCT displays a broad spectrum of antimicrobial activity, including Gram-positive and Gram-negative bacteria, chlamydiae and fungi [14]. Octenidine is well-tolerated by skin-forming cells [15]. A new group of antiseptic agents has recently been more and more often used in the clinical setting to treat chronic wounds. These agents are chlorine-based and -releasing agents (sodium hypochlorite (NaClO), hypochlorous acid (HClO) and hypochlorite (OCl-)) [16]. Their antimicrobial mechanism of action relies on strong oxidative properties of the above-mentioned compounds, which lead to microbial amino acid and phospholipid degradation and hydrolysis. It has been shown that hypochlorite/hypochlorous acid solution acts efficiently against vegetative bacteria, bacterial spores, and aspergilli [17]. Other reports have revealed very low cytotoxicity of hypochlorite/hypochlorous acid agents, which increased the interest of clinical practitioners in this class of antiseptics [18]. A body of evidence concerning the efficacy of PHMB and OCT antiseptics against wound biofilm has been presented in numerous reports [19,20]. In turn, a relatively scant number of reports has been provided with regard to NaClO/HClO agents in this matter [21]. Therefore, the aim of this paper was to compare the antibiofilm efficacy of PHMB, OCT vs. NAClO/HClO-based antiseptic, using a broad range of microbial methods. The three species of biofilm-forming pathogens were chosen for analysis, namely methicillin-resistant *Staphylococcus aureus*, *Pseudomonas aeruginosa* and yeast-like fungus referred to as *Candida albicans*. The *S. aureus* and *P. aeruginosa* are pathogens, the presence of which is associated with severe infections of chronic wounds. Their appearance requires careful management because of these microbes’ ability to acquire antibiotic resistance, ability to destroy tissue and risk of infection development throughout the patient’s body [22]. In turn, *C. albicans* is considered crucial component of prevalent fungal communities responsible for delay of wound healing [23].

## 2. Materials and Methods

### 2.1. Antiseptics and Strains Analyzed

The tested antiseptic solutions were:

(a) Prontosan Wound Irrigation Solution (B. Braun Medical AG), later referred to as “P”, which contained 0.1% undecylenamidopropyl betaine, 0.1% polyhexamethylene biguanide (polihexanide), and purified water.
(b) Octenilin Wound Irrigation Solution, (Schülke Mayr GmbH, Vienna, Austria), later referred to as “O”, which contained Aqua valde purificata, Glycerol, Ethylhexylglycerin and Octenidine HCl.
(c) Microdacyn 60 Wound Care Solution (Sonoma Pharmaceuticals, Inc, Petaluma, CA, USA), later referred to as “M” which contained super-oxidized water, sodium chloride (0.022%), hypochlorous acid (0.004%), sodium hypochlorite (0.004%).

All antiseptics were provided by B. Braun Medical AG in non-transparent, unsigned bottles containing 250 mL of colorless solutions referred to as “WIS1, WIS2, WIS3”, where “WIS” stood for “wound irrigation solution”. The identity of WIS’s was decrypted after the end of research and introduced to the manuscript of this work.

### 2.2. The Following Microbial Strains of PORT [PORT Polish Center for Technology Development/Polski Ośrodek Rozwoju Technologii] Microbiology Laboratory Strain Collection Were Used:

(A) *Candida albicans* PRT1-9 [n = 9]
(B) *Pseudomonas aeruginosa* PRT1-9 [n = 9]
(C) Methicillin-Resistant *Staphylococcus aureus* (MRSA) PRT1-9 [n = 9]

The above-listed strains were isolated from chronic leg ulcers of various etiology and are part of PORT Collection of Microbial Species. For all strains, biofilm culturing was performed at 37 °C to provide temperature conditions reflecting these occurring within human body. The above-mentioned temperature is within temperature range appropriate for growth of all tested microbial species.

### 2.3. Evaluation of Minimal Biocidal Concentrations of Tested Antiseptics Using Microtiter Plate Assay

The technique was performed as presented in our earlier work [24]. Briefly: to evaluate the impact of P, O and M solutions on microbial growth, 100 μL of Mueller-Hinton (BioCorp, Warsaw, Poland) broth in case of bacteria and RPMI with 2% glucose in case of *C. albicans* was placed into the wells of 96-well test plates (Gibco, Carlsbad, CA, USA). Next, 100 µL of the specific antiseptic was added to the well. Subsequently, geometric dilutions of individual antiseptics were performed. In the next step, 100 µL of microbial suspension at a density of 1 × 10^5^ CFU/mL (established by densitometer Densitomat II, BioMerieux, Poland and subsequently by serial dilutions) was placed into the wells of 96-well test plates (Biofil, Warsaw, Poland). According to this methodology, the highest obtained concentration of the antiseptic was 25% (*v/v*). Next, the absorbance of the suspensions was measured using a spectrometer (PerkinElmer, EnSpire Multimode Plate Reader, Waltham, MA, USA) at 580 nm wavelength. Subsequently, the plates were incubated for 24 h at 37 °C in a shaker Lab Companion IST-3075R (Imgen Technologies, Alexandria, VA, USA) to obtain optimal conditions for microbial planktonic growth and to decrease the level of biofilm formation. After incubation, the absorbance value was measured once again. The medium without microbes constituted a negative control, whereas the medium with microbes and no antiseptic solutions added was used as a positive control of microbial growth. Moreover, if the spectrometric assay indicated complete growth inhibition, the well content was aseptically transferred onto Brain-Heart Infusion (BioCorp, Warsaw, Poland) agar plates and cultured for 24 h at 37 °C. If no microbial colonies occurred after that time, the suspension from the well was considered microbiologically sterile. In parallel, another experimental setting in 96-well plates was used to evaluate the MBC value. In this setting, 1% tetrazolium chloride (TTC, Sigma-Aldrich, Darmstadt, Germany) was added to the microbial suspensions previously exposed to antiseptic solutions. Next, the plates were left for 2 h at 37 °C. During this time, the survived microorganisms changed colorless TTC into red formazan. All settings were performed in three replicates. The MBC value was considered valid, if cohesive results from the first and the second setting were obtained.

### 2.4. Evaluation of Minimal Biofilm Eradication Concentration [MBEC] of Antiseptics Using Microtiter Plate Assay

This technique was performed as presented in our previous paper [24]. Briefly: a total of 100 μL of microbial suspensions at a density of 1 × 10^5^ CFU/mL was transferred into the wells of 96-well test plates and left for 24 h at 37 °C. After incubation, non-adhered and loosely adhered cells were removed by aspiration and the wells were rinsed 3× times with 0.9% NaCl. Next, geometric solutions of antiseptics in sterile medium were introduced to the plates’ wells. According to this methodology, the highest obtained concentration of the antiseptic was 50% (v/v). The plates were left for 24 h at 37 °C. After incubation, the solutions were removed and fresh medium supplemented with 1% TTC was added, and the plates were left for 2 h at 37 °C. During this time, living microorganisms changed colorless TTC into red formazan. A lack of color change in the first well next to the red-colored well showed MBEC value. The experiments were performed in three replicates.

### 2.5. Biofilm-Oriented Antiseptic Test

This technique was performed according to the methodology described in [25]. Briefly: the microbial strains (*S. aureus* PRT1-9; *C. albicans* PRT1-9; *P. aeruginosa* PRT1-9) were cultured in BHI liquid medium and incubated at 37 °C for 24 h. After incubation, the microbial suspension was diluted with fresh medium to reach 1×10^5^ cells/mL. Subsequently, 3× 100 μL of the suspension (1×10^5^ cells) of an individual microbial strain was transferred to three adjacent wells of a 96-well polystyrene plate. This procedure was performed in duplicate (plate A and plate B). Next, the suspensions were incubated at 37 °C for 24 h. After 24 h, the suspensions from both plates were removed and thoroughly rinsed using 0.9% NaCl. Next, (plate A) 100 μL of antiseptics (undiluted working solution) was transferred to the well for a selected contact time (1 min, 15 min, 30 min, 1 h, 24 h). After the contact time, the antiseptics were removed and the wells were filled with a universal neutralizing agent (Saline Peptone Water, Biocorp, Warsaw, Poland) for 5 min. Effective neutralization of chemical biocide is the first step in the accurate evaluation of antiseptics and disinfectants to avoid overestimation of the biocide activity [26]. After this time, the neutralizing agent was removed. The wells were filled with 100 μL of an appropriate medium and with 5 μL of TTC. The results were assessed colorimetrically after 24 h of incubation of the plate at 37 °C. For plate B, all the stages were performed in the same manner as for plate A, except that instead antiseptics, saline was added. Plate B was used as a control of the strains’ ability to form biofilm.

### 2.6. Cellulose-Based Biofilm Model

*Komagataeibacter xylinus* PRT1 was used to produce bacterial cellulose. The strain was cultivated for 7 days in self-prepared Herstin–Schramm (HS) medium until complete formation of the cellulose carrier (CC). All CCs were obtained during a single cultivation period. Next, *K. xylinus* cells were removed from the CCs using alkaline lysis and rinsed with sterile water until pH stabilization. Cell-free CCs were kept refrigerated until further analyses. Next, 2 mL of the DMEM (Biowest, Riverside, MO, USA) medium (suspension containing 10^5^ cells/mL of murine fibroblasts L929 (ATCC, Manassas, VA, USA) was settled on the CC. Fibroblast proliferation was assessed using standard tetrazolium test every 24 h for 7 days. Subsequently, 2 mL of 10^5^ microbial CFU/mL of *C. albicans* PRT1-9, *S. aureus* PRT1-9, *P. aeruginosa* PRT1-9 was settled on CCs with a fibroblast layer. Half of the medium was changed every 24 h. Viability and proliferation of the cells were evaluated using quantitative culturing and tetrazolium test every 24 h for 3 days. The 24 h coculture of microbes and fibroblasts was chosen as a model for antiseptic application. Therefore, the biofilm grown on the CC was exposed to 2 mL of antiseptics for 1 h of contact time. Next, the CCs with the remaining biofilm were transferred to 10 mL of universal neutralizing agent for 5 min. After this time, the CCs with remaining biofilm were transferred to 2 mL of BHI medium containing 1% TTC and left for 2 h. Subsequently, the medium was removed, and the CCs were rinsed once again with 0.9% NaCl. Afterwards, 1 mL of formazan-extracting solution (ethanol: acetic acid 90:10 (v/v), respectively, POCH, Poland) was added to the CCs. Next, the CCs in formazan-extracting solution were mechanically vigorously shaken (vortex-mixing) for 15 min. Subsequently, the formazan solution was transferred to fresh 96-well plates and quantified at a wavelength of 490 nm using Perkin Elmer spectrometer. To assess the percentage of remaining biofilm-forming cells in the tested samples in comparison to untreated (control) samples, the following calculation was performed: Biofilm eradication (%) = 100% − (value of the test sample absorbance/value of control sample absorbance) × 100%. The CCs with pre-formed biofilm treated with saline instead of antiseptics served as a control of biofilm growth, while the CCs treated with 30% H_2_O_2_ (agent of known, strong antimicrobial efficacy) served as control of method usability. Moreover, scanning electron microscopy photographs were taken to visualize selected stages of cellulose-based biofilm model development. The samples were gently cleansed in PBS (Sigma-Aldrich, Darmstad, Germany) buffer as it was described in [27]; fixed in glutaraldehyde [28] (POCH, Wroclaw, Poland) and dried in a critical point dryer EM CPD300 (Leica Microsystems, Wetzlar, Germany). Subsequently, the samples were subjected to sputtering with Au/Pd (60:40) using EM ACE600, Leica sputter (Leica Microsystems, Wetzlar, Germany). The sputtered samples were examined using a scanning electron microscope (SEM, Auriga 60, Zeiss, Germany).

The flow charts presenting methodological aspects of performed analyses 2.3–2.6 are contained in Supplementary Data, Figures S1–S4.

### 2.7. Confocal Microscopy Examination of Chosen Biofilms Formed on CC

The biofilms of strains: *C. albicans* PRT9, *P. aeruginosa* PRT9, *S. aureus* PRT9 on CCs, exposed to antiseptics or 30% H_2_O_2_ for 1h, or non-exposed (treated with saline) were dyed with Filmtracer™ LIVE/DEAD™ Biofilm Viability Kit (Thermo Fischer Scientific, Waltham, MA, USA) according to manufacturer’s instruction. The subsequent procedures were performed analogically to these presented in [29]. Next, the materials were attached to the bottom of a Petri dish (diameter of 60 mm) employing cyanoacrylate glue (Kropelka, Uruguay, Poland). Next, approximately 2 mL of PBS buffer was added to the dish in order to cover the CCs with a buffer. Then a cover slip (18 × 18 mm, thickness 0.17 mm) was placed on the upper surface of the CC and gently pressed to remove air bubbles. The Petri dish was then placed on an SP8 confocal microscope table (Leica Microsystems, Wetzlar, Germany). A water immersion objective with 25× magnification and numerical aperture of 0.95 (HC FLUOTAR L, Leica Microsystems, Wetzlar, Germany) was used for imaging. Three randomly selected 3D fields with dimensions of 372.3 × 372.3 μm (xy axes; lateral dimensions) and between 70 and 120 μm (z axis; depth of field) were imaged. The 3D pixel size (voxel) was set to 0.298 × 0.298 × 0.566 μm. The acquired images had a resolution of 1248 × 1248 pixels in xy axes. The emission of SYTO9 (probe for discrimination of living cells) was excited with a 488 nm laser line and fluorescence ranging from 492 to 533 nm was collected, whereas the emission of propidium iodide (PI; probe for discrimination of dead cells) was excited with a 552 nm laser line and fluorescence ranging from 557 to 622 nm was collected. Sequential acquisition mode was employed in order to avoid spectral cross-talk.

### 2.8. Statistical Analysis

Calculations were performed using the GraphPad Prism version 7 software (GraphPad Co., San Diego, CA, USA). The normality of distribution was assessed by means of the D’Agostino–Pearson omnibus test. Because all values were non-normally distributed, the Kruskal–Wallis test with post-hoc Dunnett analysis were applied. The results of statistical analyses were considered significant if they produced *p*-values < 0.05.

## 3. Results

In the first stage of the experiment, we have conducted a precondition analysis of the activity of the investigated antiseptic products towards planktonic (un-bounded, un-adhered) cells of *C. albicans*, *S. aureus* and *P. aeruginosa* using a standard MBC evaluation performed in 96-well plates. Both polihexanide- and octenidine-based antiseptics (P and O, respectively) were able to kill the planktonic forms of the analyzed pathogens, even after several dozen-fold dilutions of the antiseptics’ working solution (Table 1). In turn, the NaClO/HClO (M) antiseptic was unable to inhibit the growth of the analyzed microbes within the tested concentration ranges. The highest concentration of active substance which can be applied in the MBC methodology is 25% of the antiseptic’s stock solution.

In the next step, using another methodology (referred to as the MBEC assessment), we have scrutinized the ability of the tested substances to eradicate biofilm formed in the wells of a 96-well plate. The *Candida*, *Staphylococcus* and *Pseudomonas* biofilms formed eagerly on the bottom of wells of 96-well plate (please refer to the Supplementary Materials Figure S5.)

**Figure 1:**
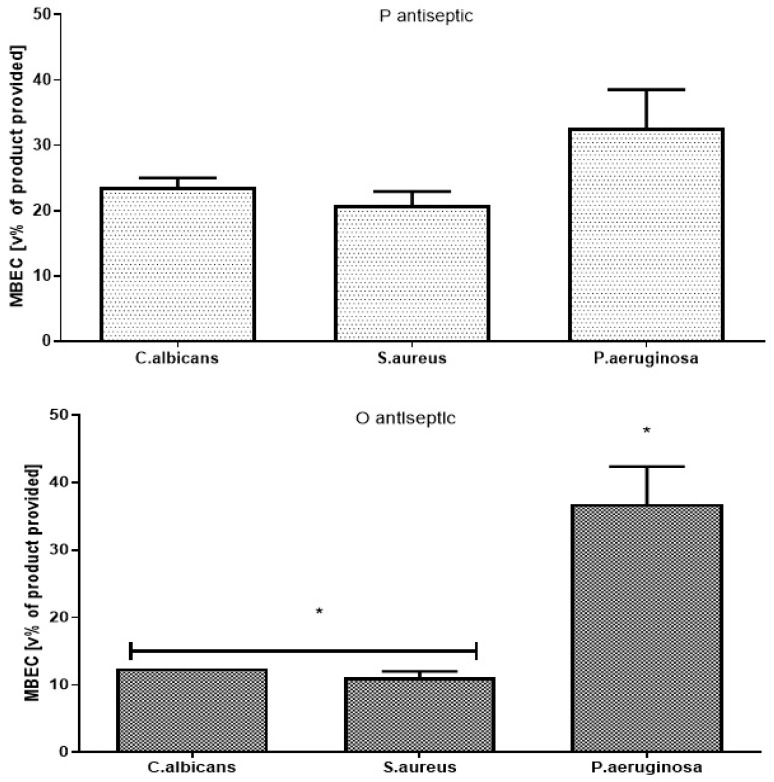
Minimal biofilm eradication concentration (MBEC) of P and O antiseptics against the tested strains (n = 9) of *C. albicans*, *S. aureus*, *P. aeruginosa*. In the case of the M antiseptic, the MBEC value for all pathogenic biofilms tested was higher than the maximum concentration (i.e., 50% of the volume of the product provided) which could be applied according to this methodology. The asterisks (*) in the bottom figure show a statistically significant difference between the ability of the O- antiseptic to eradicate *P. aeruginosa* biofilms vs. *C. albicans* and *S. aureus* biofilms; *p* < 0.05). P, O—polihexanide, octenidine dihydrochloride-based antiseptic, respectively.

It turns out that to eradicate biofilm, a few dozen times higher concentrations of antiseptics are required (Figure 1) than the concentrations of antiseptics inhibiting the growth of the same strains’ planktonic counterparts (Table 1). Nevertheless, the concentrations of P and O antiseptics required to eradicate biofilm in a 96-well plate setting were still 5× or even 10× lower than the concentrations of the relevant antiseptic’s working solutions provided in commercial products. Pseudomonal biofilm occurred to be the biggest challenge for O antiseptic and the difference in concentrations required to eradicate biofilm formed by *P. aeruginosa* was statistically significant in comparison to the setting when the O antiseptic was applied against the biofilm formed by *C. albicans* and *S. aureus* (K-W test, *p* < 0.05). A similar trend was also observed for the P antiseptic, but it was statistically insignificant. Again, in this specific methodology the highest antiseptic’s concentration possible to achieve is 50% (*v/v*) of the antiseptic’s working solution. No MBEC values were detected when the M antiseptic was applied against *C. albicans*, *P. aeruginosa* or *S. aureus* biofilms within the analyzed range of concentrations.

In the subsequent experimental setting, we have performed a biofilm-oriented antiseptic test (BOAT), which allows the analyses of the antiseptic activity in the clinically-relevant contact time and to apply undiluted antiseptics (Table 2A–C). The results indicate that none of tested antiseptics were fully effective within 1 min of contact time against the tested pathogens; none of the antiseptics applied were also effective against all *Candida albicans* biofilms within 15 min of contact time. The P and O antiseptics displayed similar levels of biofilm eradication within 30 min and 1 h of contact time against *S. aureus* and *P. aeruginosa* (Table 2B,C, respectively); in turn, the O antiseptic eradicated *C. albicans* biofilms more effectively than the P antiseptic within 1 h of contact time (Table 2A). The M antiseptic was unable to eradicate completely the biofilm formed by all tested microbial strains, even in the longest 24 h contact time.

**Figure 2:**
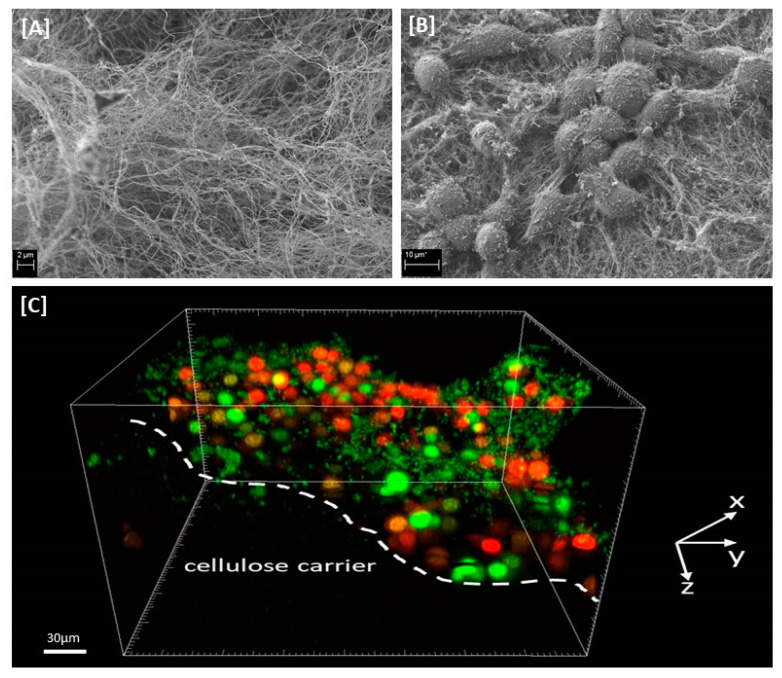
Presentation of cellulose-based biofilm model. (**A**) native cellulose-carrier (SEM, magn. 5000×); (**B**) cellulose carrier covered with fibroblasts (SEM, magn. 2500×); (**C**) staphylococcal (PRT9) biofilm formed on fibroblast-containing cellulose carrier visualized with confocal microscopy. Big red (dead) and green (live) oval shapes—fibroblasts; smaller green dots—staphylococcal cells. The figure shows a side view projection of the cellulose carrier (not visible) covered with a fibroblast layer and bacteria. A high share of dead fibroblasts (red oval shapes) during 24 h of co-culture with staphylococci is worth noting.

Finally, we have scrutinized the antibiofilm activity of the antiseptics using a self-designed cellulose-based biofilm model. Similarly to the BOAT, it allows the application of clinically-relevant contact times and working solutions of antiseptics. Moreover, it utilizes cellulosic biopolymer as an elastic and porous surface (Figure 2A) for the growth of skin fibroblasts (Figure 2B) cocultured with microbial biofilm (Figure 2C).

**Figure 3:**
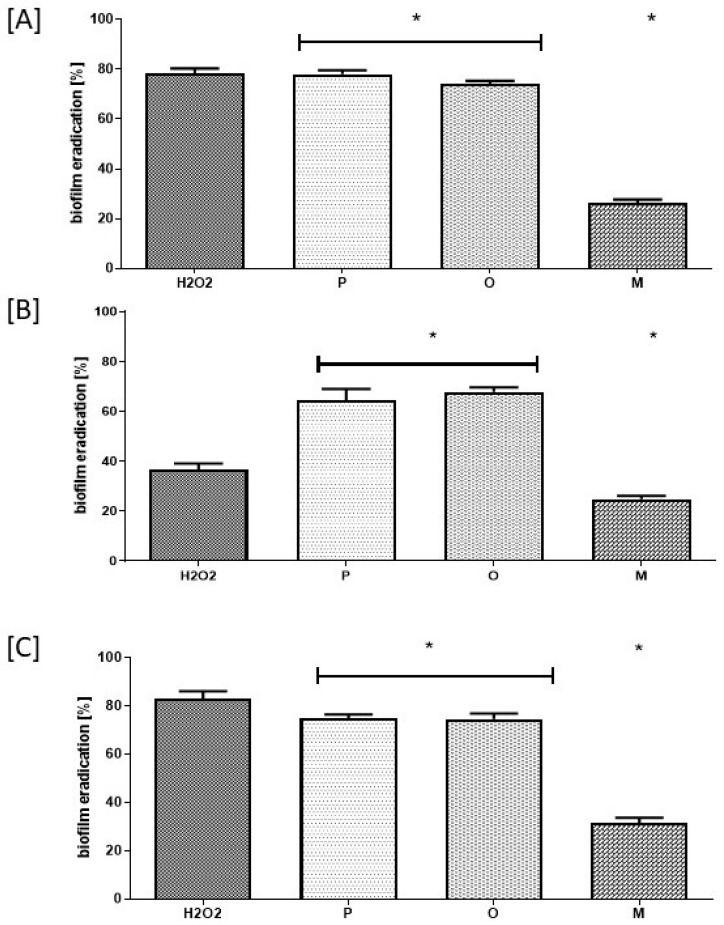
Eradication of (**A**) *C. albicans*; (**B**) *S. aureus*, (**C**) *P. aeruginosa* biofilm from fibriblast-covered cellulose carrier by P, O and M antiseptics. P, O, M—polihexanide, octenidine dihydrochloride, NaClO/HClO-based antiseptics, respectively. H_2_O_2_ of concentration 30% was used as a control of microbial killing, while the number of biofilm-forming cells immersed in saline were considered 100% of potential microbial viability. The asterisks (*) represent statistical significance (*p* < 0.05) between individual antiseptics’ activity.

**Figure 4:**
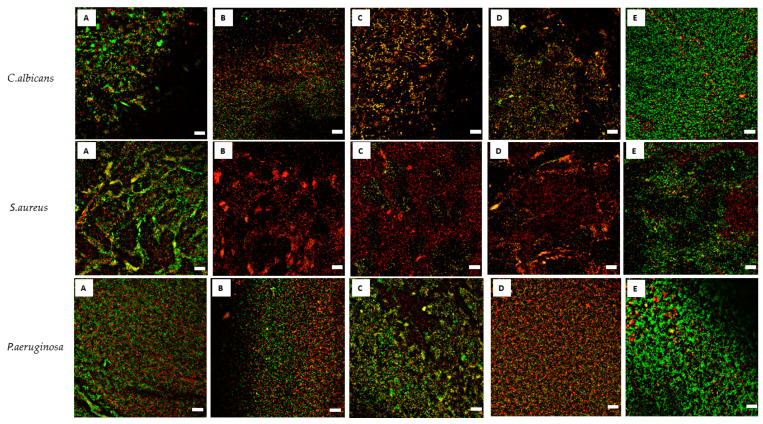
Live/dead images of biofilm-forming cells forming on a cellulose carrier subjected to the antiseptics for 1 h. (**A**) untreated biofilm (no antimicrobial solution); (**B**) 30% H_2_O_2_, biofilm treated with 30% hydrogen peroxide, i.e., compound of strong antimicrobial activity; (**C**) biofilm treated with P antiseptic; (**D**) biofilm treated with O antiseptic; (**E**) biofilm treated with M antiseptic. Green—live cells, red/orange—dead cells. The white bars in the right lower part of every picture are of 50 µm of longitude. Please also refer to supplementary data Figures S6 and S7 where pictures of bigger size with characters and typical points are presented.

The results of the analysis utilizing cellulose-based biofilm model (Figure 3) have shown that within 1 h of contact time, both P and O antiseptics displayed a similar ability to eradicate (~75%) *C. albicans*, *S. aureus* and *P. aeruginosa* biofilm-forming cells. In comparison to P and O antiseptics, a statistically significantly lower ability (~20%) to kill biofilm-forming cells of the aforementioned pathogens was displayed by M antiseptic. The results of this parametric analysis were additionally confirmed by confocal microscopy (Figure 4). As can be observed, the intensity of green color (confirming the presence/number of living microorganisms) is in the case of M antiseptic (Figure 4E) comparable to the untreated control (Figure 4A), while the strong reduction of the green color’s intensity and the increase of the red color intensity (dyeing dead cells) is seen when 30% H_2_O_2_, P and O antiseptic are applied (Figure 4B–E, respectively).

## 4. Discussion

An effective antiseptic, used to treat colonized/infected chronic wounds should display anti-biofilm properties. It means that such antimicrobial should be able to penetrate through the extracellular matrix and to kill biofilm-forming cells [11,30]. Therefore, in the first line of investigation we have analyzed the impact of P, O and M antiseptics on so called planktonic (un-adhered) cells of *C. albicans*, *S. aureus* and *P. aeruginosa* (Table 1). It has to be pointed out here, that using this methodology the maximum achievable concentration of antiseptic product was 25% (*v/v*) of the so-called “working solution”, i.e., the concentration of the antiseptic which is provided by its manufacturer for use. Nevertheless, the P and O antiseptics displayed values of minimal biocidal concentrations even when diluted a few dozen times below the “working solution” threshold (Table 1). The P and O antiseptics displayed similar activity against the tested *S. aureus* strains; the O antiseptic was more efficient against *C. albicans* than the P antiseptic. Such results stay in line with the reports showing high anti-yeast potential of the earlier-mentioned antiseptic [19]. The *P. aeruginosa* cells were the most tolerant against P and O antiseptics, although both antimicrobials were able to kill the cells of this and all other tested pathogens even when highly diluted. It should also be stressed that *P. aeruginosa* possesses a high tendency to form slime. Even in a planktonic state it forms aggregates which are frequently removed from experimental settings during subsequent cycles of rinsing and pipette-based aspiration [31]. It results in relatively high standard deviations in outcomes as was observed also in the data presented in this paper. Still, the P and O antiseptics displayed high antipseudomonal activity which could be assessed within this particular experimental setting. Contrary to that, no MBC values were assessed for the M antiseptic within the tested range of concentrations (Table 1). Total chlorine content is considered to be the key physicochemical property of the M antiseptic with regard to its antimicrobial efficacy. The M antiseptic contains 80 ppm (parts per million) of this element. Assuming that the entire chlorine content was successfully released from the HOCl/NaOCl compounds of the M antiseptic, its highest concentration in the discussed experimental setting was 20 ppm. It satisfactorily explains the lack of observed antimicrobial effect, corresponding to the recent report of Severing et al. [32], who showed no antimicrobial effect of undiluted M antiseptic against *S. aureus* and *P. aeruginosa* after 15 min. of contact time. Only NaClO/HClO antiseptics with high content of chlorine (>670 ppm) displayed antimicrobial efficacy in aforementioned study. Another factor contributing to no microbicidal effect of the M antiseptic may be related to long contact time applied in MBC assessment, i.e., 24 h. Unlike polihexanide and octenidine, chlorine displays no remanence effect [11], therefore a relatively low number of survived microorganisms could multiply during the aforementioned period of incubation, which resulted in no MBC value observed after 24 h. In the subsequent analysis, also utilizing a 96-well plate format, we scrutinized the antibiofilm activity of P, O and M antiseptics. Due to, indicated by Bueno et al. [33], significant methodological inaccuracies of standard biofilm biomass assessment in aforementioned setting, we focused on estimation of microbial viability after exposure on antiseptics rather than on evaluation of drop of biofilm biomass. The results presented in Figure 1 indicate that biofilm structure provides microbial cells significantly (a few dozen times) higher tolerance to antiseptics than that displayed by their planktonic counterparts (please compare results of Figure 1 and Table 1), which stays in line with the generally-recognized protective function of biofilm structure [34]. Similarly to the results presented for planktonic cells in Table 1, also the *P. aeruginosa* biofilm-forming cells displayed the highest tolerance to P and O antiseptics; ~40–50% concentration of their working solutions had to be applied to eradicate the biofilm formed by all 9 tested pseudomonal strains. Nevertheless, both the P and O antiseptics were highly active against biofilms formed by the three types of wound pathogens. Similarly to the data presented in Table 1 concerning planktonic cells, the M antiseptic showed no measurable activity against *C. albicans*, *S. aureus* and *P. aeruginosa* biofilms. In this experimental model, the maximum achievable concentration of this antiseptic was 50% of the provided solution so it contained 40 ppm of chlorine. Yet, it has to be once again noted that the cells within biofilm were a few dozen times more tolerant to P and O antiseptics than their planktonic counterparts. It thus seems rational that if the 20 ppm of chlorine was ineffective against planktonic cells, the chlorine’s doubled (to 40 ppm) concentration could not be efficient against highly-tolerant biofilm structures. Presently, the local application of antibiotics to the infected wound is not-recommended by the major European Societies of Wound Healing due to possible adverse effects and risk of resistance occurrence; one of the few exceptions is local application of gentamycin antibiotic coupled with collagen carrier (garamycin sponge). The gentamycin displays high efficacy against susceptible *S. aureus* and *P. aeruginosa* strains, however the increasing percentage of resistant strains of aforementioned species lowers applicability of this biomaterial. Moreover, the concentrations of antibiotic able to eradicate microbial biofilms are relatively high. Maczynska et al. [35] indicated that high local concentrations of gentamicin released from collagen sponge eradicated the biofilm formed not only by gentamicin-sensitive strains but, to some extent, also by these that display a resistance pattern in routine diagnostics. Nevertheless, values of effective concentrations of gentamycin were in aforementioned Maczynska’s work above 100 mg/L in case of *P. aeruginosa* and *S. aureus* biofilm and above 700 mg/L in case of *Klebsiella pneumoniae*. Thanks to unspecific mechanism of action, effective concentrations of P and O antiseptics, recorded in this study, were a few times lower in comparison to gentamycin (Figure 1). The knowledge concerning biofilm and biofilm testing is a relatively fresh branch of science. That is why, a vast number of techniques (including normative protocols) designed to evaluate antimicrobial efficacy display certain disadvantages with regard to biofilm-related analysis. It applies to some extent also to the above-presented (Figure 1) method of MBEC evaluation. Therefore, there has been an ongoing process within the scientific world to establish new models for in vitro biofilm testing. One of such models, referred to as the biofilm-oriented antiseptic test (BOAT) has been developed by our team [25]. BOAT allows the analyses of the antibiofilm efficacy of antiseptics within clinically relevant contact times and using working solutions of antiseptics. Still, the BOAT results presented in Table 2 stay in line not only with the screening analyses presented in Table 1 and Figure 1 but also with the aforementioned work by Severing et al. [32] who showed that the M antiseptic was ineffective against *S. aureus* and *P. aeruginosa* within a contact time of 1–15 min. Our results, presented in Table 2, show inefficacy of the M antiseptic also in 30 and 60 min of contact time. One should however note that the analysis of Severing was performed for only 1–15 min and against planktonic microbial cells. Taking into consideration the fact of the already indicated (Figure 1) high tolerance of biofilm-forming cells against the M antiseptic and the rapid mode of chlorine’s antimicrobial action [36], the results presented in Table 2., showing a sustained lack of M antiseptic efficacy within 30 and 60 min after exposure, stay in line with the data presented by other research teams. Interestingly, the O antiseptic was more effective against *C. albicans* than the P antiseptic also in this experimental model, although in a long, 30 min contact time. Both of these antiseptics were unable to fully eradicate *C. albicans* biofilm within shorter, 1 min and 15 min, contact times. The prolonged contact time between the antiseptic (or any other antimicrobial) and the wound may potentially correlate with the broad spectrum of adverse effects (from cytotoxic effects toward fibroblasts to risk of development of microbial resistance). To avoid such a hazard, the use of antiseptics against highly tolerant microorganisms should be coupled with surgical operations and dressings application, providing more complex care on chronic wound. As the clinical studies show that both P and O antiseptics are capable of effective eradication of *Candida* biofilm, the question which should be also addressed is how relevant biofilm cultured in in vitro settings is to the actual wound biofilm. Indeed, an increasing number of reports show that the in vitro biofilm grows in too favorable conditions (with regard to temperature, access to nutrition, lack of immune system) [37]. It can lead to results showing that their tolerance to antiseptics is higher than it would otherwise be within an actual patient’s chronic wound. Therefore, in the last experimental setting, we have scrutinized the activity of the tested antiseptics using the self-developed biocellulose-based biofilm model (Figure 2). Our aim was to provide in vitro conditions more resembling those found in actual chronic wounds. To reach this goal we have applied a spongy biocellulose mesh as a surface for cellular growth (instead of polystyrene surface used in the majority of other in vitro biofilm-oriented tests) and we have settled on it a fibroblast cell layer and, subsequently, with biofilm formed by the tested pathogens. The results presented earlier by our team [38] indicated that the porous, fibrinous scaffold of biocellulose (Figure 2A,B) may be an excellent environment for microbial cell development, while Loh et al. have shown that biocellulose also displays excellent properties with regard to attachment of such eukaryotic cells as wound-healing fibroblasts [39]. Therefore, we have scrutinized activity of P, O and M antiseptics against biofilm co-cultured with fibroblasts on the biocellulose scaffold (Figure 2C). The results presented in Figure 3. are coherent with the results shown in Table 1, Figure 1 and Table 2 of this manuscript with regard to comparable activities of P and O antiseptics against the tested pathogens. However, contrary to the results from the aforementioned polystyrene plate-based assays indicating a lack of antibiofilm activity of the M antiseptic, data obtained in the biocellulose-based model have shown that this antimicrobial was able to eradicate ~20% of biofilm formed by *C. albicans*, *S. aureus* and *P. aeruginosa*. Nevertheless, P and O antiseptics’ antibiofilm activity against all tested biofilms was still statistically significantly higher (*p* < 0.05) than the antibiofilm activity of the M antiseptic. It should also be mentioned that even the P and O antiseptics were unable to fully eradicate biofilm from the biocellulose-based model. It was already reported that microbes are capable of penetrating through the cellulosic membrane [38]. It may be assumed that the biofilm hidden beneath the cellulosic fibers could be additionally protected from the antiseptic activity analogically as in the case of actual wound biofilm developed beneath the wound clot or within wound pockets and topologic irregularities. The parametric data presented in Figure 3 were additionally backed up by the analysis performed using confocal microscopy (Figure 4) showing vast areas of damaged biofilms after exposure to P and O antiseptic and only slightly-altered (in comparison to the growth control setting, Figure 3A) surfaces treated with the M antiseptic. It should be mentioned that the data concerning the application of NaOCl/HOCl in clinical settings for infected/colonized wound treatment is extremely scanty. One of the few reports on the matter in question is the clinical randomized trial by Assadian et al. [21], concerning the use of wet-to-moist cleansing with different irrigation solutions to reduce bacterial bioburden in chronic wounds. In this study, which covered 260 patients with 299 chronic wounds, the highest reduction of bacterial burden was achieved with an aqueous solution containing betaine, zinc and polihexanide; followed by 3% saline solution containing 0.2% sodium hypochlorite. It should be noted that in the discussed work, the applied concentration of hypochlorite was 0.2%, while the chlorine content in the M antiseptic was 0.008% (25 times lower concentration); moreover, the chlorine-based antiseptic applied by Assadian et al. contained also a hypertonic concentration of 3% saline (while the M antiseptic contains only 0.022% of NaCl); which is a compound of known mucolytic properties [40] and may disrupt biofilm aggregates, freeing the cells from the protective matrix coating and making them more susceptible to flushing out and to the antiseptic agent. Thus, the data presented by Assidian et al. stay in line with the already-mentioned report [32] showing a lack of antimicrobial activity of NaOCl/HOCl antiseptics containing total chlorine content from 80–100 ppm and proving the antimicrobial activity of these of NaOCl/HOCl antiseptics whose chlorine content was >670 ppm. It should be stressed here that the reports of other teams show a lack of cytotoxicity of NaOCl/HOCl antiseptics with a chlorine content of 80–100 ppm [11] against skin-forming and wound-forming cells. In analysis performed by Severing et al. [32], the application of the same type of M antiseptic as the one used in this study, had no negative impact of wound fibroblasts (BJ cells) and keratinocytes within time of 1–15 min and no cytotoxic effect was detected. However, NaOCl/HOCl antiseptics with chlorine content >670 ppm displayed cytotoxic effect against fibroblasts already within 5 min. of contact time. In turn, the high tolerability of wound fibroblasts to P and O antiseptics within contact time of 30 min was indicated by Muller et al. [15], giving a strong back-up for application of these antiseptics in clinical practice. Therefore, in light of the results presented here, M antiseptic may be applied to rinse the wound and to provide it the moist environment required for proper healing [41]. However, their use should be carefully considered with regard to the specific patient and his wound status, if strictly antimicrobial procedures are to be applied. In such circumstances, in our opinion, the application of other classes of antiseptic agents should be considered. We are aware of certain limitations of our study. First of all, because of the reported high inter- and intra-species variability with regard to specific antimicrobial measures, a bigger number of microbial strains should be scrutinized to provide a more comprehensive picture of the analyzed antiseptics’ activity [42]. Therefore, drawing on the experience gained in this research, we are planning to conduct a broad screening analysis including at least two hundred microbial strains isolated from chronic wounds of various etiology and confront them with a wide range of antiseptics, including chlorhexidine, povidone-iodine and ethacridine lactate.

Secondly, we are aware of the specific disadvantages [31] of plate-based in vitro settings we have applied (which can have an impact on the outcome obtained) and of the fact that biofilms cultured in laboratory conditions may do not fully resemble these inhabiting a patient’s wound. We have tried to eliminate these disadvantages by applying a differentiated methodology and to gain various perspectives on the same phenomena observed. Still, one should be careful with direct translating the results obtained in this research to the clinical setting.

Thirdly, our biocellulose biofilm model would more resemble actual wound environment if it was enriched with certain components of immune system (both cellular and non-cellular), however introduction and standardization of this additional factor to our model, exceeded scope of this research. Nevertheless, we believe that the data presented in this report, together with the results of other research teams, may be helpful in choosing the appropriate antiseptic for critically colonized/infected chronic wounds.

## 5. Conclusions

1. In vitro analysis of biofilm requires the application of diversified analytical techniques to provide cohesive results
2. PHMB and octenidine-based antiseptics displayed similar and high antimicrobial activity against biofilms formed by *C. albicans*, *S. aureus* and *P. aeruginosa* strains
3. M antiseptic, of chlorine content equals 80ppm displayed no antibiofilm activity in 2 out of 3 performed analyses and weak antibiofilm activity in cellulose-based biofilm model

## Figures and Tables

**Table 1:** Minimal Biocidal Concentration [MBC] of P, O, M antiseptics against the test strains (n = 9) of *C. albicans*, *S. aureus*, *P. aeruginosa*.

MBC (*v/v*%) of Antiseptic Working Solution			
(n = 9)	*C. albicans*	*S. aureus*	*P. aeruginosa*
P	0.36 (±0.18)	0.17 (±0.03)	1.56 (±0.95)
O	0.09	0.18	0.45 (±0.19)
M	>25% *	>25% *	>25% *

The asterisk shows that MBC for M was beyond the highest concentration (25% *v/v*) of the antiseptic applied. P, O, M—Polihexanide, Octenidine dihydrochloride, NaClO/HClO—based antiseptic, respectively.

**Table 2:** Ability of the tested antiseptics to eradicate biofilm of (A) *C. albicans*, (B) *S. aureus* and (C) *P. aeruginosa* measured by biofilm-oriented antiseptic test. P, O, M—plihexanide, octenidine dihydrochloride, NaClO/HClO-based antiseptics, respectively.

**(A) *Candida albicans* (n = 9).**	**Contact Time**				
	**1 min**	**15 min**	**30 min**	**1 h**	**24 h**
	**(%) of Biofilm-Forming Strains Survived Treatment**				
P antiseptic	100	100	100	66.6	0
O antiseptic	100	100	66.6	0	0
M antiseptic	100	100	100	100	100
**(B) *S. aureus* (n = 9).**	**Contact Time**				
	**1 min**	**15 min**	**30 min**	**1 h**	**24 h**
	**(%) of Biofilm-Forming Strains Survived Treatment**				
P antiseptic	100	77.7	77.7	66.6	0
O antiseptic	100	66.6	55.5	55.5	0
M antiseptic	100	100	100	100	100
**(C) *P. aeruginosa* (n = 9).**	**Contact Time**				
	**1 min**	**15 min**	**30 min**	**1 h**	**24 h**
	**(%) of Biofilm-Forming Strains Survived Treatment**				
P antiseptic	100	77.7	77.7	66.6	0
O antiseptic	100	66.6	55.5	55.5	0
M antiseptic	100	100	100	100	100

## Data Availability

The data presented in this study are available on request from the corresponding author as the dataset obtained is planned to be applied in subsequent, chemometric analyses.

## Supplementary Materials

The following are available online at https://www.mdpi.com/2077-0375/11/1/62/s1, Figure S1: Flow chart of Minimal Biocidal Concentration performance; TTC—tetrazolium chlo-ride. Figure S2: Flow chart of Minimal Biofilm Eradication Concentration performance; TTC—tetrazo-lium chloride. Figure S3: Flow chart of Biofilm-Oriented Antiseptic Test performance; TTC—tetrazolium chlo-ride. Figure S4: Flow chart of assessment using Cellulose-Based Biofilm Model; TTC—tetrazolium chlo-ride. Figure S5: The Cristal Violet-dyed biofilm formed by *S. aureus* PRT-9, *P. aeruginosa* PRT-9, *C. albicans* PRT-9 on the bottom of polystyrene well. Figure S6: The untreated with antiseptics (positive control of growth) staphylococcal biofilm dyed with Live/Dead stain-ing kit. Figure S7: Staphylococcal biofilm treated with O-antiseptic for 1h and dyed with Live/Dead staining kit.
