# Supplementary material for: In Vitro Evaluation of Polihexanide, Octenidine and NaClO/HClO-Based Antiseptics against Biofilm Formed by Wound Pathogens
Source: Membranes (Basel). 2021 Jan 17;11(1):62. doi: 10.3390/membranes11010062 (PMC7830887; doi:10.3390/membranes11010062)
Supplement: Supplementary file 1 [file membranes-11-00062-s001.pdf]

membranes

MDPI

Supplementary Files

In Vitro Evaluation of Polihexanide, Octenidine and NaClO/HClO - Based Antiseptics Against Biofilm Formed by Wound Pathogens

Grzegorz Krasowski[1], Adam Junka[2,3*], Justyna Paleczny[2], Joanna Czajkowska[3], Elżbieta Makomaska-Szaroszyk[4], Grzegorz Chodaczek[5], Michał Majkowski[5], Paweł Migdał[6], Karol Fijałkowski[7], Beata Kowalska-Krochmal[2] and Marzenna Bartoszewicz[2]

[1] Nutrikon, KCZ Surgical Ward, 47-300 Krapkowice and Poland; g.krasowski@wp.pl
[2] Department of Pharmaceutical Microbiology and Parasitology, Faculty of Pharmacy Wrocław Medical University, 50-556 Wrocław, Poland; justyna.paleczny@student.umed.wroc.pl, marzen-na.bartoszewicz@umed.wroc.pl; beata.kowalska-krochmal@umed.wroc.pl; adam.junka@umed.wroc.pl
[3] Laboratory of Microbiology, Łukasiewicz Research Network–PORT Polish Center for Technology Development, 54-066 Wrocław, Poland; joanna.czajkowska@port.org.pl; adam.junka@port.org.pl
[4] Faculty of Medicine, Lazarski University, 02-662Warszawa, Poland; elzbieta.makomaska-szaroszyk@lazarski.pl
[5] Bioimaging Laboratory, Łukasiewicz Research Network–PORT Polish Center for Technology Development, 54-066 Wrocław, Poland; grzegorz.chodaczek@port.org.pl; michal.majkowski@port.org.pl
[6] Department of Environment Hygiene and Animal Welfare, Wroclaw University of Environmental and Life Sciences, 25 C.K. Norwida st., 51-630 Wroclaw, Poland; pawel.migdal@upwr.pl
7 Department of Microbiology and Biotechnology, Faculty of Biotechnology and Animal Husbandry, West Pomeranian University of Technology, 70-311 Szczecin, Poland; karol.fijalkowski@zut.edu.pl
* Correspondence: whom correspondence should be addressed: Adam Junka, Medical University of Wrocław, Faculty of Pharmacy with Medical Analytics Division, Department of Pharmaceutical Micro-biology and Parasitology, Borowska 211, 50-556 Wrocław, Poland. feliks.junka@gmail.com, tel. no: +48 71 784 06 75

Citation: Krasowski, G.; Junka, A.; Paleczny, J.; Czajkowska, J.; Elżbieta Makomaska-Szaroszyk4; Chodaczek, G.; Michał Majkowski5; Paweł Migdał6; Fija, K.; Kowalska-Krochmal, B.; et al. In Vitro Evaluation of Polihexanide, Octenidine and NaClO/HClO - Based Antiseptics Against Biofilm Formed by Wound Pathogens. *Membranes* **2021**, *11*, x. https://doi.org/10.3390/xxxxx

Received: date
Accepted: date
Published: date

Publisher's Note: MDPI stays neutral with regard to jurisdictional claims in published maps and institutional affiliations.

Copyright: © 2021 by the authors. Submitted for possible open access publication under the terms and conditions of the Creative Commons Attribution (CC BY) license (http://creativecommons.org/licenses/by/4.0/).

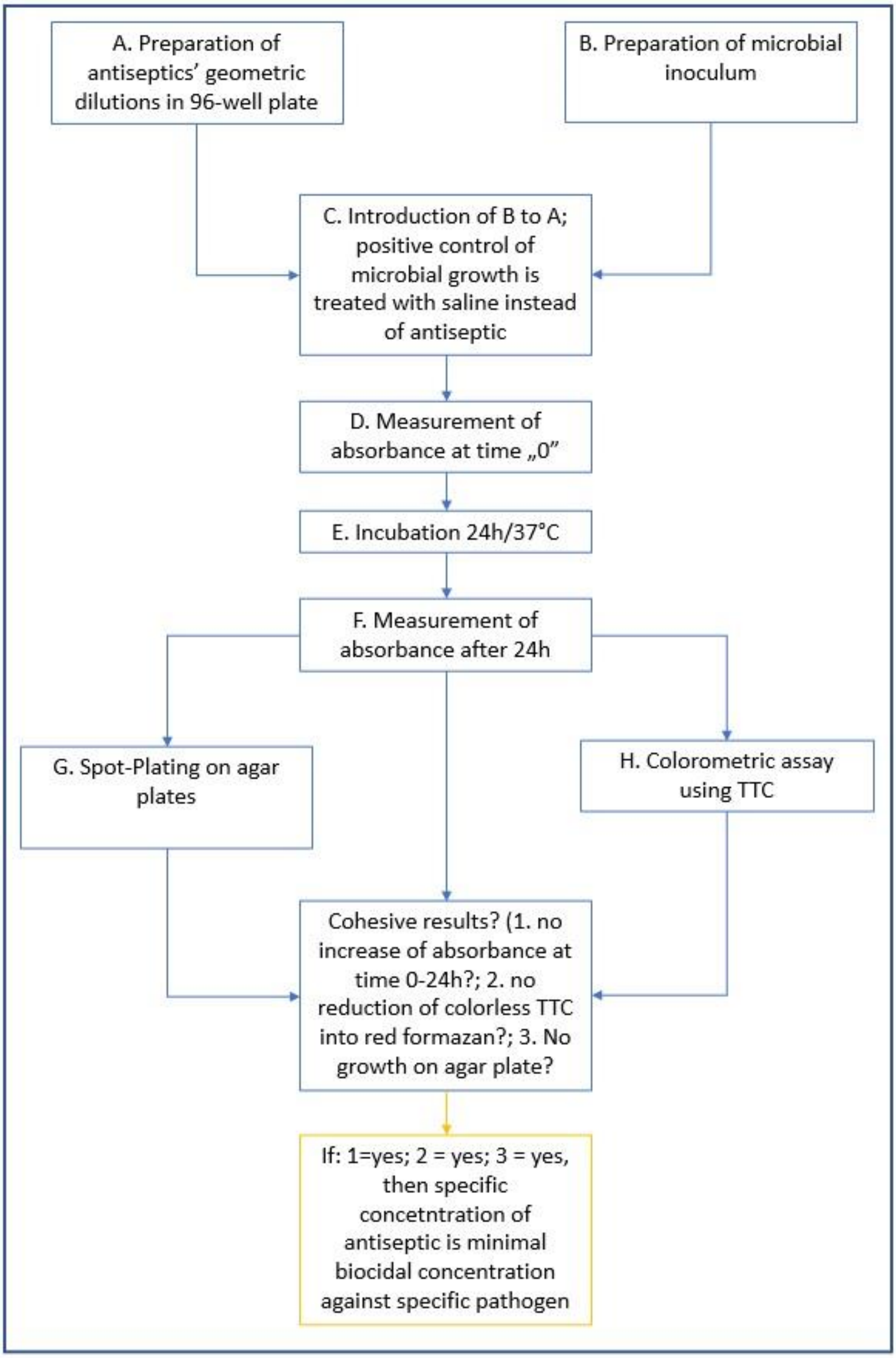

Figure S1. Flow chart of Minimal Biocidal Concentration performance; TTC – tetrazolium chloride.

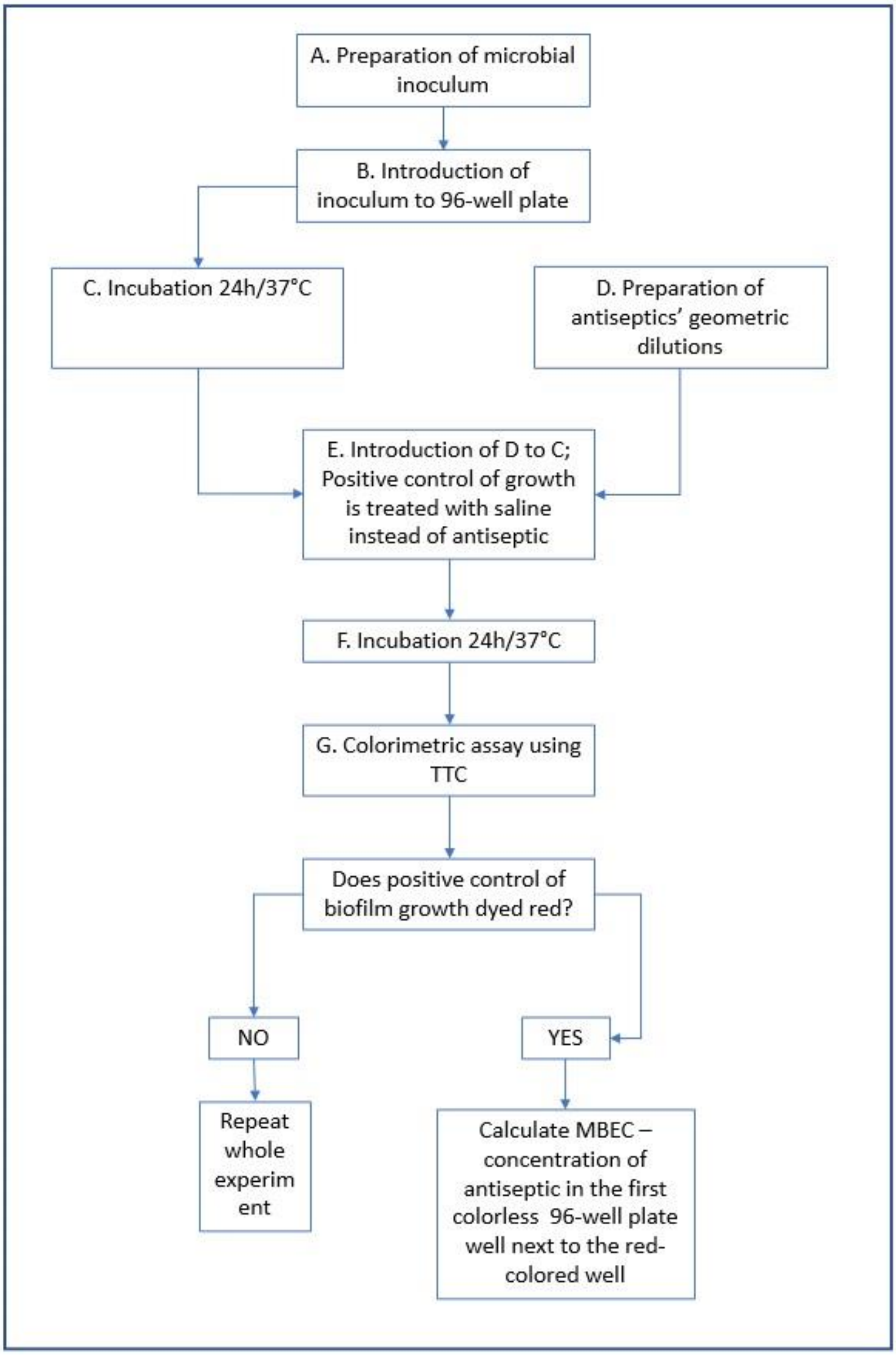

Figure S2. Flow chart of Minimal Biofilm Eradication Concentration performance; TTC – tetrazolium chloride.

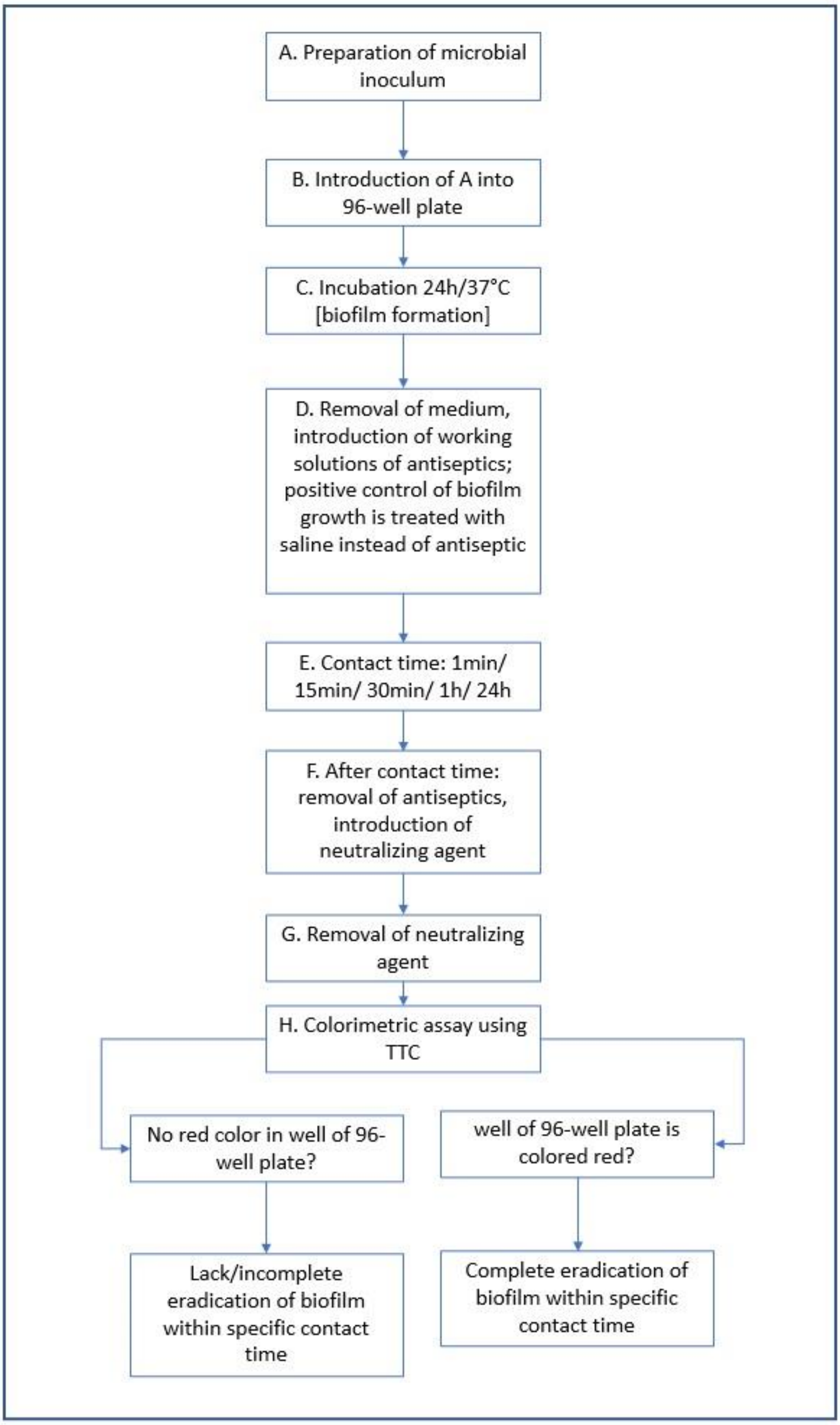

Figure S3. Flow chart of Biofilm-Oriented Antiseptic Test performance; TTC – tetrazolium chloride.

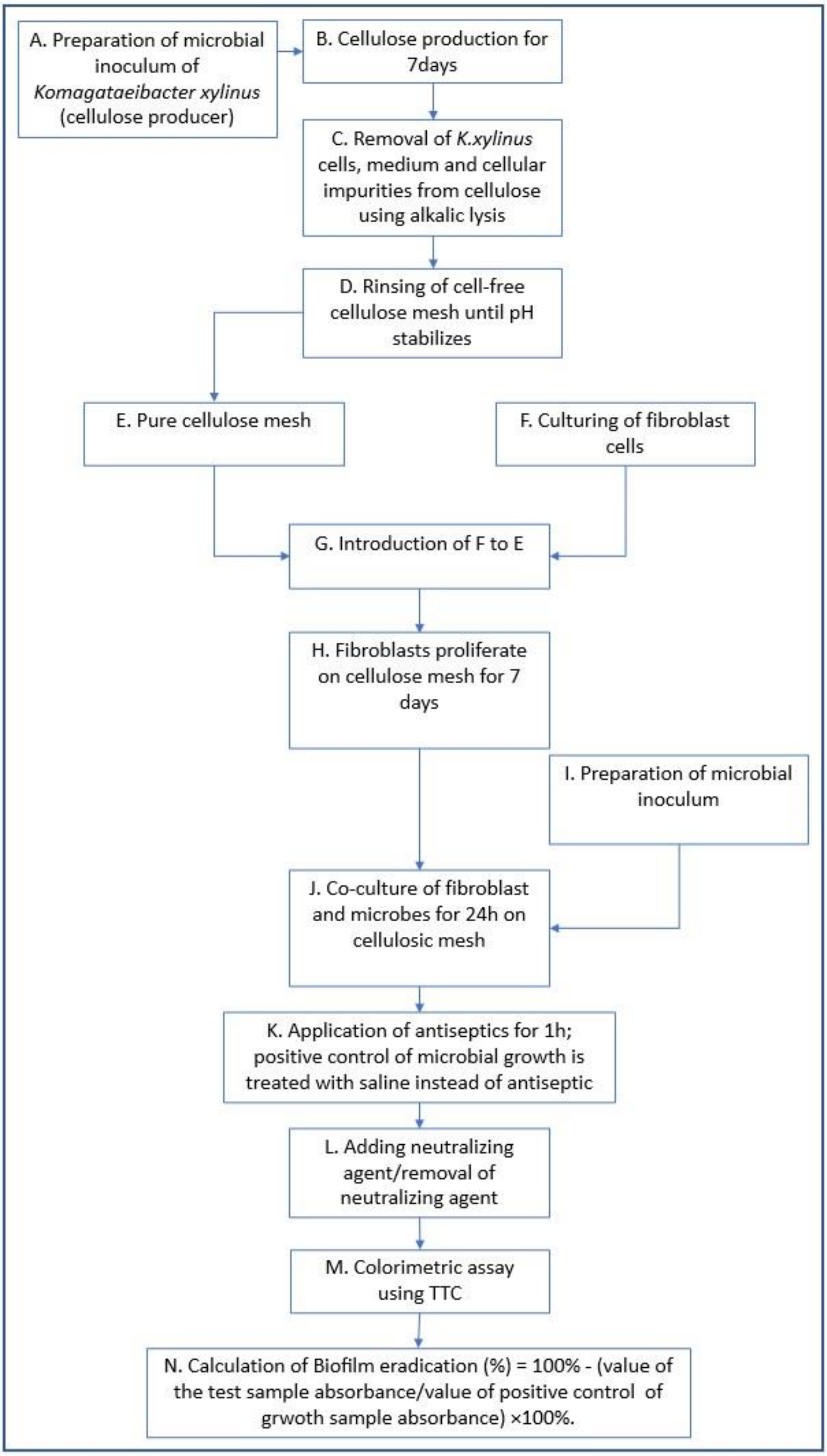

Figure S4. Flow chart of assessment using Cellulose-Based Biofilm Model; TTC – tetrazolium chloride.

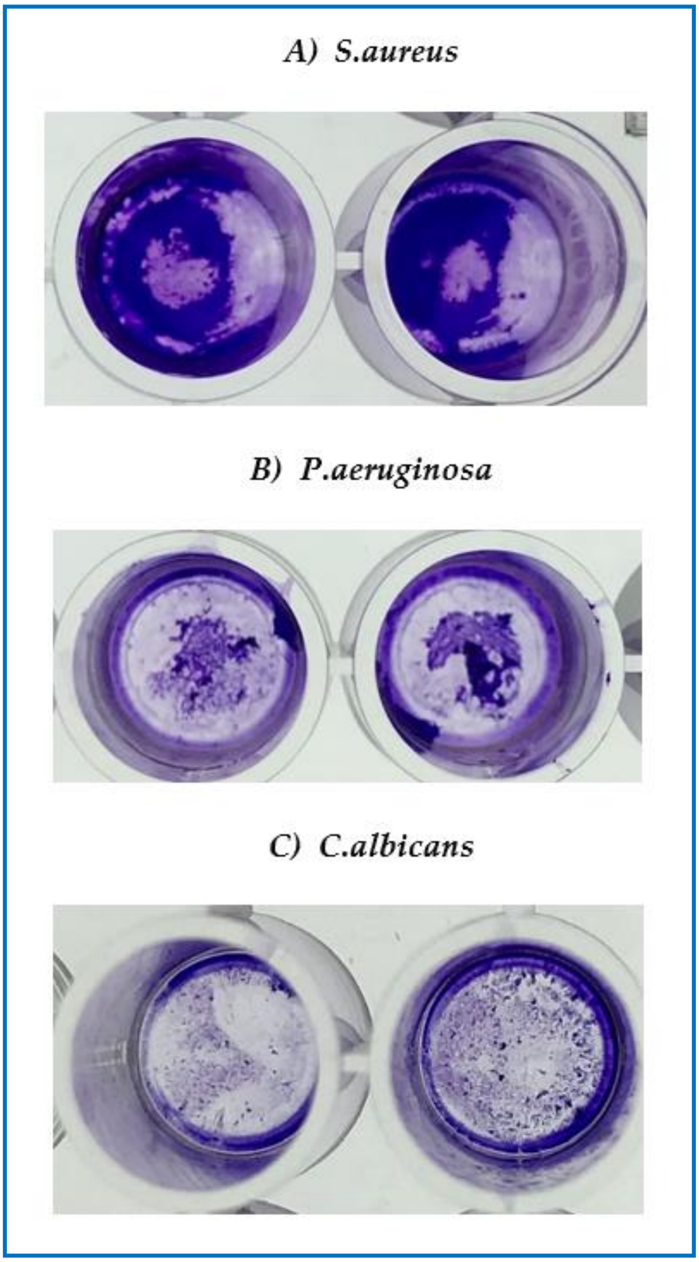

Figure S5. The Cristal Violet-dyed biofilm formed by *S.aureus* PRT-9, *P.aeruginosa* PRT-9, *C.albicans* PRT-9 on the bottom of polystyrene well. Please note that majority of biofilm biomass is located on the flat surface of well. The standard Crystal Violet technique for biofilm dyeing was performed according to the protocol presented by Merritt JH, Kadouri DE, O'Toole GA. *Growing and analyzing static biofilms*. Curr Protoc Microbiol. 2005;Chapter 1:Unit-1B.1. doi:10.1002/9780471729259.mc01b01s00.

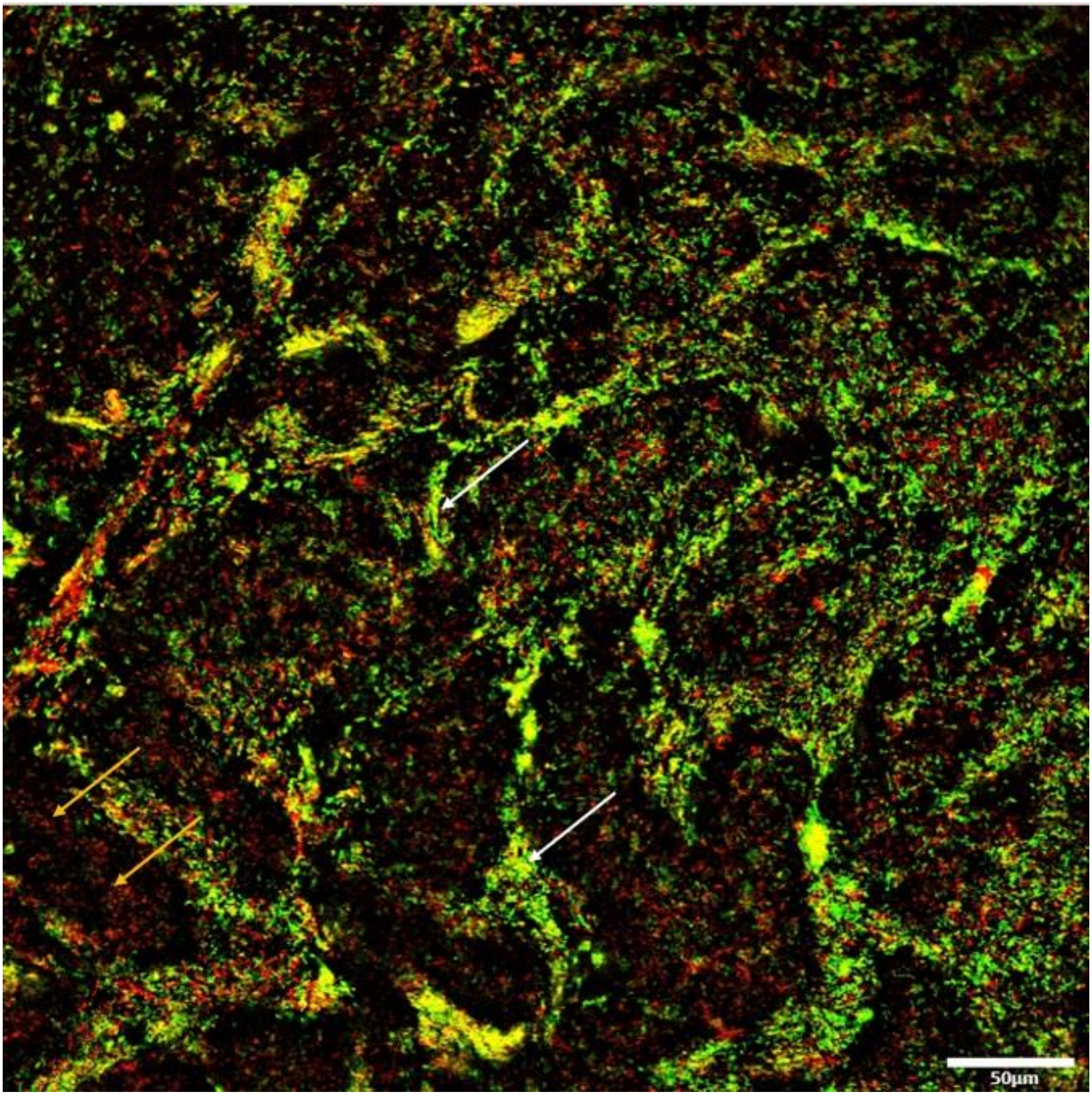

Figure S6. The untreated with antiseptics (positive control of growth) staphylococcal biofilm dyed with Live/Dead staining kit. White arrows indicate aggregates of live staphylococcal cells (dyed green), while orange arrows indicate aggregates of dead staphylococcal stains (dyed red). Please note that staphylococcal biofilm untreated with antiseptics consists of live and dead cells, however the number of live cells prevails over dead cells.

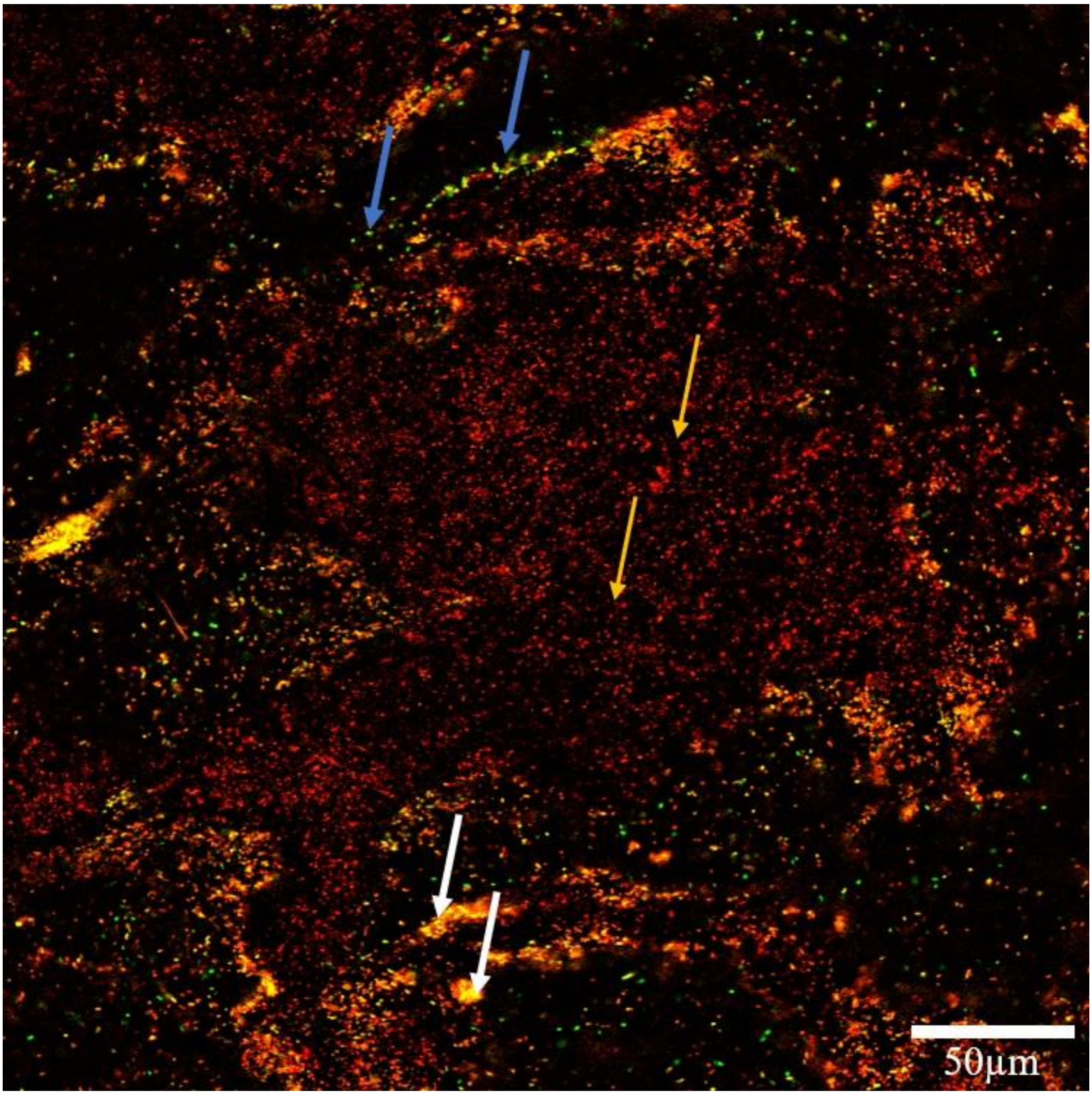

Figure S7. Staphylococcal biofilm treated with O-antiseptic for 1h and dyed with Live/Dead staining kit. Blue arrows indicate aggregates of live staphylococcal cells (dyed green); orange arrows indicate aggregates of dead staphylococcal stains (dyed red), while white arrows indicate aggregates of staphylococcla cells with partially damaged cell wall (incorporation of two dyes). Please note the higher share of black (undyed) surfaces in this picture comparing to picture presented in **Fig.S6**. These are surfaces, where complete biofilm removal occurred.
